# Hyperimmune intravenous immunoglobulin containing high titers of pandemic H1N1 hemagglutinin and neuraminidase antibodies provides dose-dependent protection against lethal virus challenge in SCID mice

**DOI:** 10.1186/1743-422X-11-70

**Published:** 2014-04-16

**Authors:** Christine Hohenadl, Walter Wodal, Astrid Kerschbaum, Richard Fritz, M Keith Howard, Maria R Farcet, Daniel Portsmouth, John K McVey, Donald A Baker, Hartmut J Ehrlich, P Noel Barrett, Thomas R Kreil

**Affiliations:** 1 Vaccine R&D, Baxter BioScience, Orth/Donau, Austria; 2 Global Pathogen Safety, Baxter BioScience, Benatzkygasse 2-6, 1221 Vienna, Austria; 3 Global Quality, Baxter BioScience, Deerfield, Illinois, USA; 4 Global R&D, Baxter BioScience, 1220 Vienna, Austria

**Keywords:** H1N1, IVIG, Influenza, Intravenous immunoglobulin, Passive transfer, Neutralizing antibody, Neuraminidase, Hemagglutinin

## Abstract

**Background:**

Convalescent plasma and fractionated immunoglobulins have been suggested as prophylactic or therapeutic interventions during an influenza pandemic.

**Findings:**

Intravenous immunoglobulin (IVIG) preparations manufactured from human plasma collected before the 2009 H1N1 influenza pandemic, and post-pandemic hyperimmune (H)-IVIG preparations were characterized with respect to hemagglutination inhibition (HI), microneutralization (MN) and neuraminidase-inhibiting (NAi) antibody titers against pandemic H1N1 (pH1N1) and seasonal H1N1 (sH1N1) viruses. The protective efficacy of the IVIG and H-IVIG preparations was evaluated in a SCID mouse challenge model.

Substantial levels of HI, MN and NAi antibodies against pH1N1 (GMTs 1:45, 1:204 and 1: 727, respectively) and sH1N1 (GMTs 1:688, 1:4,946 and 1:312, respectively) were present in pre-pandemic IVIG preparations. In post-pandemic H-IVIG preparations, HI, MN and NAi antibody GMTs against pH1N1 were 1:1,280, 1:11,404 and 1:2,488 (28-, 56- and 3.4-fold enriched), respectively, compared to pre-pandemic IVIG preparations (p < 0.001). Post-pandemic H-IVIG (HI titer 1:1,280) provided complete protection from lethality of SCID mice against pH1N1 challenge (100% of mice survived for 29 days post-challenge). Pre-pandemic IVIG (HI titer 1:70) did not provide significant protection against pH1N1 challenge (50% of mice survived 29 days post-challenge compared to 40% survival in the buffer control group). There was a highly significant correlation between circulating *in vivo* HI and MN antibody titers and survival (p < 0001).

**Conclusion:**

The substantial enrichment of HA- and NA-specific antibodies in H-IVIG and the efficacious protection of SCID mice against challenge with pH1N1 suggests H-IVIG as a promising intervention against pandemic influenza for immunocompromised patients and other risk groups.

## Background

Infectious diseases were commonly managed by passive transfer of human or animal sera prior to the development of effective vaccines and antimicrobials, and passive transfer of convalescent sera is currently still used to prevent and treat some viral and bacterial infections [1,2]. The use of convalescent plasma and fractionated immunoglobulins [3,4], and, more recently, monoclonal antibodies [5-8], has been suggested as a complementary strategy to prevent or treat virus infection during an influenza pandemic. Treatment of severe pH1N1 infection with convalescent plasma or H-IVIG was associated with lower viral load and reduced mortality [9,10].

As previously reported [11], we investigated the feasibility of manufacturing H-IVIG as a response to the emergence of the 2009 H1N1 pandemic (pH1N1) virus. H-IVIG preparations were shown to have significantly elevated levels of pH1N1 hemagglutinating (HI) and neutralizing antibodies, as assessed by microneutralization (MN) assay, compared to standard IVIG preparations collected from donors either during or before the H1N1 pandemic [11].

In the present study, we further characterized post-pandemic H-IVIG preparations with respect to HI and MN antibodies against both pH1N1 and a seasonal H1N1 (sH1N1) virus (A/New Caledonia/20/1999, which circulated in the Northern hemisphere and was a recommended component of the trivalent seasonal vaccine from 2000–01 to 2006–07). We also assessed the level of pre-existing cross-reactive pH1N1 immunity in the donor population by determining the HI and MN titers against pH1N1 and sH1N1 in pre-pandemic IVIG preparations. In addition, we investigated the extent to which antibodies capable of inhibiting the enzymatic activity of the sH1N1 and pH1N1 neuraminidase (NA) antigens were enriched in the post-pandemic compared to pre-pandemic preparations. We also investigated the ability of pre-pandemic IVIG and post-pandemic H-IVIG to protect highly susceptible immunodeficient SCID mice against challenge with wild-type pH1N1 virus.

## Methods

### IVIG and H-IVIG

A total of 13 IVIG preparations (Gammagard Liquid/KIOVIG, Baxter Healthcare, Westlake Village, CA) manufactured from human plasma collected before the 2009 H1N1 influenza pandemic (manufactured January to November 2009, plasma collected >6 months prior to these dates), and two lots of post-pandemic H-IVIG preparations (manufactured July 2010) [11] were characterized in the present study. For IVIG production, collected plasma was pooled into two 7500-L batches, and the Cohn ethanol fractionation and downstream processes for the two H-IVIG lots were performed in full accordance with the licensed process for Gammagard Liquid/KIOVIG, a triple virus-reduced 10% IVIG preparation [12,13].

### Laboratory Assays

Pre-pandemic IVIG and H-IVIG preparations were characterized with respect to HI and MN titers against pH1N1 (A/California/07/2009 reassortant NYMC X-179A), and sH1N1 (A/New Caledonia/20/1999), as previously described [11].

Antibodies capable of inhibiting the enzymatic activity of NA (NAi antibodies) in the IVIG and H-IVIG preparations were detected using a highly sensitive enzyme-linked lectin assay (ELLA), as previously described [14]. N1 antigens were obtained by splitting wild-type A/California/07/2009 (CDC, Atlanta, GA) and A/New Caledonia/20/1999 virus preparations by overnight incubation with 0.5% Triton X-100 at 4°C, followed by detergent removal using 20% Bio-Beads (Biorad).

### Mouse passive transfer studies

6–8 week old SCID mice (strain CB17/Icr-Prkdcscid/IcrCrl; Charles River Laboratories, Germany) were intranasally infected with 30 μl containing 6×10^4^ tissue culture infectious dose 50% (TCID_50_) of H1N1 strain A/California/07/2009 (9.5-fold 50% lethal (LD_50_) dose [15]) and monitored 29 days. For passive protection studies, mice were intraperitoneally injected three days prior to challenge with 200 μl of H-IVIG, IVIG or PBS control. Two independent experiments with two different lots of H-IVIG (8–10 mice per group), and two independent experiments with a pool of 5 lots of IVIG or PBS control (10 mice per group) were done. For calculation of dose-dependency, 200 μl of undiluted and 2-fold serial dilutions of H-IVIG, undiluted IVIG or phosphate-buffered saline (PBS) were used. Serum was obtained from animals before challenge, at Day 3, and at Day 32 to determine circulating *in vivo* antibody titers.

### Statistical analysis

Statistical differences between IVIG and H-IVIG antibody titers were calculated from combined data by unpaired Student t-test analysis. Differences in survival were analyzed with a Log-rank (Mantel-Cox) test. The significance of the correlation of *in vitro* and *in vivo* HI and MN titers, as determined on Day 3 and Day 32, as well as correlation of Day 3 antibody titers with survival was evaluated using a nonparametric Spearman correlation analysis (GraphPad Prism v.5.01 software).

## Results and Discussion

**Figure 1:**
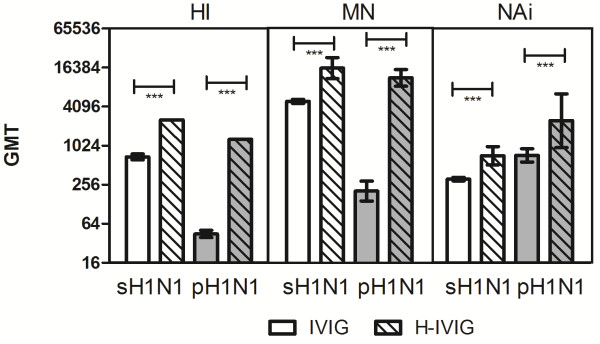
**Serological characterization of pre-pandemic IVIG (open bars) and post-pandemic H-IVIG (hatched bars).** Antibody titers against the seasonal H1N1 strain A/New Caledonia/20/1999 (sH1N1) (white bars) and pandemic A/California/07/2009 (pH1N1) (grey bars) were determined by hemagglutination inhibition (HI), microneutralization (MN) and enzyme-linked lectin assay (ELLA, reported as NA inhibition [NAi]), respectively. Results are given as geometric mean titer (GMT) ± 95% confidence interval (CI). Statistical differences were calculated by unpaired Student t-test analysis (GraphPad Prism software). *** *P* < 0.0001. GMTs were calculated by testing 13 individual lots of IVIG and 2 lots of H-IVIG.

To investigate the titers of antibodies against sH1N1 and pH1N1 in pre-pandemic (n = 13) and post-pandemic IVIG (n = 2) preparations, sera were analyzed by HI, MN and NAi assays. Figure 1 shows HI, MN and NAi titers measured in pre-pandemic IVIG preparations (open bars) and post-pandemic H-IVIG preparations (hatched bars). Pre-pandemic IVIG preparations had substantial HI, MN and NAi titers against pH1N1 (grey bars) (geometric mean titer [GMT] 1:45, 1:204 and 1:727, respectively), as well as against sH1N1 (white bars) (GMT 1:688, 1:4,946, and 1:312 respectively). As expected, significantly higher HI, MN and NAi antibody titers against pH1N1 were present in the post-pandemic H-IVIG preparations compared to titers in the pre-pandemic IVIG preparations (*P* < 0.0001 for all serological assays). GMTs of 1:2,560 (HI), 1:11,404 (MN) and 1: 2,488 (NAi) were determined for the post-pandemic H-IVIG preparations, an increase of 28- 56- and 3.4-fold, respectively, compared to pre-pandemic IVIG preparations. In addition, higher titers of HI (1:2,560), MN (1:16,127) and NAi (1:717) antibodies against sH1N1 were also present in the post-pandemic H-IVIG preparations.

The finding that substantial levels of HI and MN antibodies against pH1N1 were also present in the pre-pandemic IVIG preparations is in agreement with other vaccine and seroepidemiological studies which reported high rates of seroprotective pre-exposure pH1N1 HI and neutralizing antibody titers, likely as a result of cross-reactive antibodies induced by repeated exposure to seasonal H1N1 viruses [16]. The relatively low increase in pH1N1-specific NA antibody titers in H-IVIG likely reflects the high level of antigenic similarity between seasonal and pandemic H1N1 NA proteins [17].

**Figure 2:**
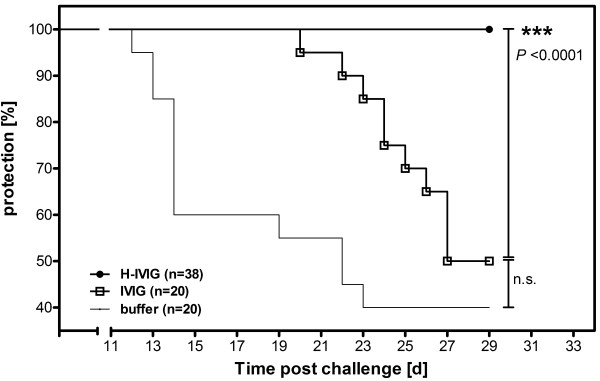
**Protective efficacy of pre-pandemic IVIG and post-pandemic H-IVIG.** To evaluate passive protection efficacy, SCID mice were intraperitoneally administered with 200 μl of pH1N1 hyperimmune IVIG (two lots, HI titer 1:1,280) or pre-pandemic IVIG (pool of five lots, HI titer 1:70) 3 days prior to challenge with 6×10^4^ TCID_50_ of wild-type pH1N1 by intranasal instillation. The control group received 200 μl of PBS and survival was monitored for 29 days. Statistical differences between the IVIG group and the PBS control group, and between the IVIG group and the H-IVIG group, were calculated with a Log-rank (Mantel-Cox) test using the GraphPad Prism software. Abbreviations: d, days; n.s., not significant; IVIG, pre-pandemic intravenous immunoglobulin; H-IVIG, hyperimmune intravenous immunoglobulin.

To analyze the protective potential of pre-pandemic IVIG and post-pandemic H-IVIG preparations, passive transfer experiments were done in SCID mice. Administration of post-pandemic H-IVIG, prior to challenge with wild-type pH1N1, provided complete protection (100% survival) of SCID mice during the 29 days monitoring period, whereas only 50% of animals receiving pre-pandemic IVIG and 40% of animals receiving buffer survived (Figure 2). Increased survival rates (compared to animals receiving pre-pandemic IVIG) afforded by post-pandemic were highly statistically significant (Mantel-Cox Log-rank *P* < 0.0001) but not for pre-pandemic IVIG compared to buffer control (*P* = 0.15). The relatively long survival time of the mice receiving buffer only is a result of the relatively low pathogenicity of the pH1N1 virus in mice [18,19]. Due to constraints of the challenge volume used, it was however not possible to increase the challenge dose.

To investigate the extent to which protection against pH1N1 challenge by H-IVIG administration is dose-dependent, mice were administered with serial 2-fold dilutions of H-IVIG prior to challenge with pH1N1. Circulating *in vivo* HI and MN antibody titers were measured in sera taken from animals immediately prior to challenge and at the end of the experiment i.e. 29 days after virus challenge. Table 1 shows the HI and MN titers of H-IVIG preparations administered to mice, the circulating antibody titers measured at the time of challenge and 29 days after challenge, and the associated survival rates of mice challenged with pH1N1 virus. Protection was dose-dependent, and there was a highly significant correlation (nonparametric Spearman correlation *r* = 0.9, *P* <0.0001) between circulating *in vivo* HI and MN antibody titers measured on the day of virus challenge and survival.

**Table 1:** Antibody titers and protection

**Sample (dilution)**	**HI GMT**			**MN GMT**			**% survival**
	**In vitro**^**a**^	**In vivo**		**In vitro**^**a**^	**In vivo**		
		**Day 3**^**b**^	**Day 32**^**c**^		**Day 3**^**b**^	**Day 32**^**c**^	
H-IVIG (undiluted)	1280	163	33	3420	349	34	100
H-IVIG (1:2)	640	143	26	1710	190	15	95
H-IVIG (1:4)	320	63	14	855	95	11	90
H-IVIG (1:8)	160	35	8	428	48	6	76
H-IVIG (1:16)	80	21	6	214	24	5	63
IVIG (undiluted)	70	6	7	60	5	5	50
Buffer control	n.a.	5	5	n.a.	5	5	40

^a^*in vitro* titer of neat samples determined prior to i.p. injection; titers of diluted H-IVIG are calculated accordingly.

^b^mouse serum titers 3 days after passive transfer; i.e. at the time of challenge.

^c^mouse serum titers 32 days after passive transfer; i.e. 29 days after challenge.

HI, hemagglutination inhibition; MN, microneutralization; GMT, geometric mean titer; n.a., not applicable.

Note: IVIG was a pooled preparation from 5 different IVIG lots; 2 different lots of H-IVIG were used separately. Data shown are combined data from 2 independent experiments.

Although these data demonstrate the potential of H-IVIG as a potential prophylactic intervention in the event of an influenza pandemic, there are several limitations to our study. In addition to showing the protective effect of H-IVIG with respect to survival of challenged animals, it would have been interesting to investigate the ability of H-IVIG to ameliorate disease symptoms (e.g. by prevention of weight loss) and to reduce viral load and cytokine levels in challenged animals. It would also have been interesting to determine whether post-pandemic H-IVIG also protects against influenza viruses of other subtypes. Several studies have reported that infection or vaccination with pH1N1 boosted broadly neutralizing HA stem antibodies in humans [20-24].

An additional limitation is that we did not investigate the potential therapeutic efficacy of H-IVIG. Several recent studies have demonstrated that post-infection administration of influenza-specific monoclonal antibodies can effectively treat mice or ferrets which were previously subjected to virus challenge [5-8]. Treatment of humans with severe pH1N1 infection with convalescent plasma or H-IVIG was also associated with lower viral load and reduced mortality [9,10]. However, prophylactic passive administration of H-IVIG could be administered to acutely immunocompromised individuals who are at high-risk of serious complications resulting from influenza infection and who may not mount an effective response to vaccination, such as HIV patients, transplant recipients and cancer patients. In the event of a pandemic caused by a highly pathogenic influenza virus, passive immunization might also be considered for first-line health care workers and for direct contacts of infected individuals.

## Conclusion

Taken together, the data in this study demonstrating substantial enrichment of HA- and NA-specific antibodies in H-IVIG and the efficacious protection of SCID mice against challenge with pH1N1 indicate that H-IVIG could be used as a prophylactic intervention against pandemic influenza for immunocompromised patients and other risk groups.

## Competing interests

All authors are employees of Baxter BioScience, manufacturer of the pandemic H1N1 hyperimmune intravenous immunoglobulin. WW, AK, MKH, JKMcV, DAB, HJE, PNB and TRK have stock options. The study was funded by Baxter BioScience.

## Authors’ contributions

CH, WW, MKH, JKM, DAB and TRK conceived and planned the study. CH, WW; AK, RF and MRF collected the data. All authors were involved in data analysis and drafting and revision of the manuscript. All authors read and approved the final manuscript.

## References

[B1] Luke TC Casadevall A Watowich SJ Hoffman SL Beigel JH Burgess TH Hark back: passive immunotherapy for influenza and other serious infections Crit Care Med 2010 38 e66 e73 20154602 10.1097/CCM.0b013e3181d44c1e

[B2] Keller MA Stiehm ER Passive immunity in prevention and treatment of infectious diseases Clin Microbiol Rev 2000 13 602 614 10.1128/CMR.13.4.602-614.2000 11023960 PMC88952

[B3] World Health Organization WHO Blood Regulators Network - Position Paper on collection and Use of Convalescent Plasma or Serum as an Element in Pandemic Influenza Planning 2009 Geneva, Switzerland: WHO 1 4 http://www.who.int/bloodproducts/brn/BRNPosition-ConvPlasma10July09.pdf

[B4] Leider JP Brunker PA Ness PM Convalescent transfusion for pandemic influenza: preparing blood banks for a new plasma product? Transfusion 2010 50 1384 1398 20158681 10.1111/j.1537-2995.2010.02590.x PMC7201862

[B5] Friesen RH Koudstaal W Koldijk MH Weverling GJ Brakenhoff JP Lenting PJ Stittelaar KJ Osterhaus AD Kompier R Goudsmit J New class of monoclonal antibodies against severe influenza: prophylactic and therapeutic efficacy in ferrets PLoS One 2010 5 e9106 10.1371/journal.pone.0009106 20161706 PMC2817000

[B6] Nguyen HH Tumpey TM Park HJ Byun YH Tran LD Nguyen VD Kilgore PE Czerkinsky C Katz JM Seong BL Song JM Kim YB Do HT Nguyen T Nguyen CV Prophylactic and therapeutic efficacy of avian antibodies against influenza virus H5N1 and H1N1 in mice PLoS One 2010 5 e10152 10.1371/journal.pone.0010152 20405007 PMC2854139

[B7] Prabhu N Prabakaran M Hongliang Q He F Ho HT Qiang J Goutama M Lim AP Hanson BJ Kwang J Prophylactic and therapeutic efficacy of a chimeric monoclonal antibody specific for H5 haemagglutinin against lethal H5N1 influenza Antivir Ther 2009 14 911 921 10.3851/IMP1413 19918095

[B8] Simmons CP Bernasconi NL Suguitan AL Mills K Ward JM Chau NV Hien TT Sallusto F Ha DQ Farrar J De J Lanzavecchia A Subbarao K Prophylactic and therapeutic efficacy of human monoclonal antibodies against H5N1 influenza PLoS Med 2007 4 e178 10.1371/journal.pmed.0040178 17535101 PMC1880850

[B9] Hung IF To KK Lee CK Lee KL Chan K Yan WW Liu R Watt CL Chan WM Lai KY Koo CK Buckley T Chow FL Wong KK Chan HS Ching CK Tang BS Lau CC Li IW Liu SH Chan KH Lin CK Yuen KY Convalescent plasma treatment reduced mortality in patients with severe pandemic influenza A (H1N1) 2009 virus infection Clin Infect Dis 2011 52 447 456 10.1093/cid/ciq106 21248066 PMC7531589

[B10] Hung IF To KK Lee CK Lee KL Yan WW Chan K Chan WM Ngai CW Law KI Chow FL Liu R Lai KY Lau CC Liu SH Chan KH Lin CK Yuen KY Hyperimmune IV immunoglobulin treatment: a multicenter double-blind randomized controlled trial for patients with severe 2009 influenza A(H1N1) infection Chest 2013 144 464 473 10.1378/chest.12-2907 23450336

[B11] Kreil TR Mc Vey JK Lei LS Camacho L Wodal W Kerschbaum A Segura E Vandamme E Gavit P Ehrlich HJ Barrett PN Baker DA Preparation of commercial quantities of a hyperimmune human intravenous immunoglobulin preparation against an emerging infectious disease: the example of pandemic H1N1 influenza Transfusion 2012 52 803 809 21981280 10.1111/j.1537-2995.2011.03347.x

[B12] Poelsler G Berting A Kindermann J Spruth M Hammerle T Teschner W Schwarz HP Kreil TR A new liquid intravenous immunoglobulin with three dedicated virus reduction steps: virus and prion reduction capacity Vox Sang 2008 94 184 192 10.1111/j.1423-0410.2007.01016.x 18167162

[B13] Teschner W Butterweck HA Auer W Muchitsch EM Weber A Liu SL Wah PS Schwarz HP A new liquid, intravenous immunoglobulin product (IGIV 10%) highly purified by a state-of-the-art process Vox Sang 2007 92 42 55 10.1111/j.1423-0410.2006.00846.x 17181590

[B14] Fritz R Sabarth N Kiermayr S Hohenadl C Howard MK Ilk R Kistner O Ehrlich HJ Barrett PN Kreil TR A vero cell-derived whole-virus H5N1 vaccine effectively induces neuraminidase-inhibiting antibodies J Infect Dis 2012 205 28 34 10.1093/infdis/jir711 22090447

[B15] Kistner O Crowe BA Wodal W Kerschbaum A Savidis-Dacho H Sabarth N Falkner FG Mayerhofer I Mundt W Reiter M Grillberger L Tauer C Graninger M Sachslehner A Schwendinger M Bruhl P Kreil TR Ehrlich HJ Barrett PN A whole virus pandemic influenza H1N1 vaccine is highly immunogenic and protective in active immunization and passive protection mouse models PLoS One 2010 5 e9349 10.1371/journal.pone.0009349 20186321 PMC2826398

[B16] Broberg E Nicoll A Mato-Gauci A Seroprevalence to influenza A(H1N1) 2009 virus–where are we? Clin Vaccine Immunol 2011 18 1205 1212 10.1128/CVI.05072-11 21653743 PMC3147351

[B17] Marcelin G DuBois R Rubrum A Russell CJ McElhaney JE Webby RJ A contributing role for anti-neuraminidase antibodies on immunity to pandemic H1N1 2009 influenza A virus PLoS One 2011 6 e26335 10.1371/journal.pone.0026335 22039464 PMC3200314

[B18] Belser JA Wadford DA Pappas C Gustin KM Maines TR Pearce MB Zeng H Swayne DE Pantin-Jackwood M Katz JM Tumpey TM Pathogenesis of pandemic influenza A (H1N1) and triple-reassortant swine influenza A (H1) viruses in mice J Virol 2010 84 4194 4203 10.1128/JVI.02742-09 20181710 PMC2863721

[B19] Kalthoff D Grund C Harder TC Lange E Vahlenkamp TW Mettenleiter TC Beer M Limited susceptibility of chickens, turkeys, and mice to pandemic (H1N1) 2009 virus Emerg Infect Dis 2010 16 703 705 10.3201/eid1604.091491 20350393 PMC3321957

[B20] Li GM Chiu C Wrammert J McCausland M Andrews SF Zheng NY Lee JH Huang M Qu X Edupuganti S Mulligan M Das SR Yewdell JW Mehta AK Wilson PC Ahmed R Pandemic H1N1 influenza vaccine induces a recall response in humans that favors broadly cross-reactive memory B cells Proc Natl Acad Sci U S A 2012 109 9047 9052 10.1073/pnas.1118979109 22615367 PMC3384143

[B21] Miller MS Tsibane T Krammer F Hai R Rahmat S Basler CF Palese P 1976 and, H1N1 influenza virus vaccines boost anti-hemagglutinin stalk antibodies in humans J Infect Dis 2009 2013 207 98 105 10.1093/infdis/jis652 PMC3523798 23087428

[B22] Qiu C Huang Y Wang Q Tian D Zhang W Hu Y Yuan Z Zhang X Xu J Boosting heterosubtypic neutralization antibodies in recipients of 2009 pandemic H1N1 influenza vaccine Clin Infect Dis 2012 54 17 24 10.1093/cid/cir753 22052887 PMC3243653

[B23] Sangster MY Baer J Santiago FW Fitzgerald T Ilyushina NA Sundararajan A Henn AD Krammer F Yang H Luke CJ Zand MS Wright PF Treanor JJ Topham DJ Subbarao K B cell response and hemagglutinin stalk-reactive antibody production in different age cohorts following 2009 H1N1 influenza virus vaccination Clin Vaccine Immunol 2009 2013 20 867 876 10.1128/CVI.00735-12 PMC3675965 23576673

[B24] Wrammert J Koutsonanos D Li GM Edupuganti S Sui J Morrissey M McCausland M Skountzou I Hornig M Lipkin WI Mehta A Razavi B Del RC Zheng NY Lee JH Huang M Ali Z Kaur K Andrews S Amara RR Wang Y Das SR O'Donnell CD Yewdell JW Subbarao K Marasco WA Mulligan MJ Compans R Ahmed R Wilson PC Broadly cross-reactive antibodies dominate the human B cell response against 2009 pandemic H1N1 influenza virus infection J Exp Med 2011 208 181 193 10.1084/jem.20101352 21220454 PMC3023136

